# Effects of cardioactive drugs on human induced pluripotent stem cell derived long QT syndrome cardiomyocytes

**DOI:** 10.1186/s40064-016-1889-y

**Published:** 2016-02-29

**Authors:** Jukka Kuusela, Ville J. Kujala, Anna Kiviaho, Marisa Ojala, Heikki Swan, Kimmo Kontula, Katriina Aalto-Setälä

**Affiliations:** BioMediTech, University of Tampere, Finn-Medi 5, Biokatu 12, 33014 Tampere, Finland; School of Engineering and Applied Science, Wyss Institute for Biologically Inspired Engineering, Harvard University, Boston, MA USA; Department of Medicine, University of Helsinki, Helsinki, Finland; School of Medicine, University of Tampere, Tampere, Finland; Heart Center, Tampere University Hospital, Tampere, Finland

**Keywords:** Induced pluripotent stem cell, Patient-specific, Long QT syndrome, Cardiomyocytes, Multielectrode array, Cardioactive drug, Arrhythmia

## Abstract

Human induced pluripotent stem cells (hiPSC) have enabled a major step forward in pathophysiologic studies of inherited diseases and may also prove to be valuable in in vitro drug testing. Long QT syndrome (LQTS), characterized by prolonged cardiac repolarization and risk of sudden death, may be inherited or result from adverse drug effects. Using a microelectrode array platform, we investigated the effects of six different drugs on the electrophysiological characteristics of human embryonic stem cell-derived cardiomyocytes as well as hiPSC-derived cardiomyocytes from control subjects and from patients with type 1 (LQT1) and type 2 (LQT2) of LQTS. At baseline the repolarization time was significantly longer in LQTS cells compared to controls. Isoprenaline increased the beating rate of all cell lines by 10–73 % but did not show any arrhythmic effects in any cell type. Different QT-interval prolonging drugs caused prolongation of cardiac repolarization by 3–13 % (cisapride), 10–20 % (erythromycin), 8–23 % (sotalol), 16–42 % (quinidine) and 12–27 % (E-4031), but we did not find any systematic differences in sensitivity between the control, LQT1 and LQT2 cell lines. Sotalol, quinidine and E-4031 also caused arrhythmic beats and beating arrests in some cases. In summary, the drug effects on these patient-specific cardiomyocytes appear to recapitulate clinical observations and provide further evidence that these cells can be applied for in vitro drug testing to probe their vulnerability to arrhythmia.

**Electronic supplementary material:**

The online version of this article (doi:10.1186/s40064-016-1889-y) contains supplementary material, which is available to authorized users.

## Background

Inherited long QT syndrome (LQTS) is a potentially severe arrhythmic disease that affects the electrical repolarization of the myocardium and manifests as an abnormally long QT interval on electrocardiogram (ECG) recordings. LQTS may result in polymorphic ventricular tachycardias known as torsades de pointes (TdP), which can ultimately lead to life-threatening ventricular fibrillation and sudden cardiac death (Schwartz et al. 2013).

Inherited forms of LQTS typically result from mutations in cardiac ion channel coding genes. In the case of LQT type 1 (LQT1), the mutations are present in the *KCNQ1* gene which encodes the α-subunit of the slow component of the delayed rectifier potassium current (K_v_7.1) (Wang et al. 1996), whereas LQT2 is caused by mutations of the *KCNH2* gene which encodes the α-subunit of the rapid component of the delayed rectifier potassium current (K_v_11.1) (Sanguinetti et al. 1995). Four founder mutations in these two genes have been found to explain the high (0.4 %) prevalence of LQTS in the Finnish population (Marjamaa et al. 2009). The most prevalent of these founder mutations is the C-terminal *KCNQ1* G589D missense mutation, resulting in a dysfunctional assembly domain and preventing the formation of fully functional potassium channel tetramers (Piippo et al. 2001). Another prevalent but apparently milder founder mutation, the *KCNH2* R176W missense mutation, resides in the N-terminus of the channel and accelerates the deactivation of the I_Kr_ tail current, thus leading to reduced potassium efflux (Fodstad et al. 2006). Research on the exact effects of these mutations on the functional properties of cardiomyocytes has been hampered by the lack of suitable techniques that can adequately recapitulate the full spectrum of the disease in vitro.

Human induced pluripotent stem cell (hiPSC) technology has recently made it possible to produce human cell lines with characteristics similar to those of embryonic stem cells from somatic cells of various origins (Takahashi et al. 2007; Yu et al. 2007). To date, multiple disease-specific hiPSC lines have already been established (Wu and Hochedlinger 2011). Several studies have focused on LQTS using the hiPSC technology (Bellin et al. 2013; Itzhaki et al. 2011; Kiviaho et al. 2015; Lahti et al. 2012; Matsa et al. 2011, 2014; Moretti et al. 2010). These studies suggest that hiPSC technology may be useful for modeling LQTS in vitro. Until now, large-scale drug effects have been studied with human embryonic stem cell-derived cardiomyocytes (hESC-CMs) and wild-type hiPSC-CMs (Braam et al. 2010; Caspi et al. 2009; Harris et al. 2013; Mehta et al. 2013; Navarrete et al. 2013; Yokoo et al. 2009). In this study, we investigated the effect of six clinically used drugs in control hESC- and wild-type hiPSC-CMs and in hiPSC-derived LQT-CMs. Additional value will be gained by studying drug responses at clinically relevant concentrations (Schulz and Schmoldt 2003), since many of the mutation carriers can have a latent, subclinical form of LQTS that may be exacerbated upon pharmacological challenge (Roden 2004).

We created hiPSC lines from the dermal fibroblasts of patients with *KCNQ1* G589D and *KCNH2* R176W mutations. We investigated how these patient-specific hiPSCs respond to pharmacological challenges in comparison with cardiomyocytes derived from control hESCs and hiPSCs.

## Methods

### Patient-specific pluripotent stem cell induction

The LQT1- and 2-specific hiPSCs were induced as previously described (Takahashi et al. 2007) from fibroblasts of patients carrying the *KCNQ1* (G589D) or the *HERG* (R176W) mutation (Fodstad et al. 2004) (see Additional file 1: Materials and methods). The study has been approved by Pirkanmaa Hospital District ethical committee (R08070). Details of the hiPSC lines are listed in Table 1. The subjects volunteered for the study gave their consent for skin biopsy to be taken.

**Table 1:** Details of the control and patient-specific pluripotent stem cell lines used for drug testing

Cell line	Line type	Mutation	Disease	Affected current	Source	Purpose
H7 (hESC)	hESC	Wild type	None	None	ICM	hESC control
UTA.00112.hFF (WTa)	hiPSC	Wild type	None	None	hFF	Control
UTA.01006.WT (WTb)	hiPSC	Wild type	None	None	hADF	Adult control
UTA.00208.LQT1^θ,^*^,s^	hiPSC	G589D	LQT1	I_Ks_	hADF	Symptomatic, patient-specific
UTA.00211.LQT1^θ,^*^,s^	hiPSC	G589D	LQT1	I_Ks_	hADF	Symptomatic, patient-specific
UTA.00303.LQT1^ψ,^*^,a^	hiPSC	G589D	LQT1	I_Ks_	hADF	Asymptomatic mutation carrier-specific
UTA.00313.LQT1^ψ,^*^,a^	hiPSC	G589D	LQT1	I_Ks_	hADF	Asymptomatic mutation carrier-specific
UTA.00514.LQT2^‡,a^	hiPSC	R176W	LQT2	I_Kr_	hADF	Asymptomatic mutation carrier-specific
UTA.00525.LQT2^‡,a^	hiPSC	R176W	LQT2	I_Kr_	hADF	Asymptomatic mutation carrier-specific

*ICM* inner cell mass of blastocyst, *hFF* human foreskin fibroblast, *hADF* human adult dermal fibroblast, *hESC* human embryonic stem cell, *hiPSC* human induced pluripotent stem cell

* Siblings ^‡, θ, ψ^ clonal lines from same individual ^s^ symptomatic ^a^ asymptomatic

### Stem cell culture

The control pluripotent stem cell lines used in this study were H7 (hESC, WiCell), UTA.00112.hFF [fetal wild-type from ATCC (WT)-hiPSC], and UTA.01006.WT (WT-hiPSC derived from a healthy adult). For LQT1, 4 patient-specific hiPSC lines were used: UTA.00208.LQT1, UTA.00211.LQT1, UTA.00313.LQT1 and UTA.00303.LQT1. For LQT2 2 patient-specific hiPSC lines were used: UTA.00514.LQT2 and UTA.00525.LQT2. The pluripotent stem cells were cultured on a mouse embryonic fibroblast (MEF, Millipore) feeder cell layer in KSR medium consisting of KnockOut Dulbecco’s Modified Eagle’s Medium (KO-DMEM, Invitrogen) supplemented with 20 % KnockOut serum replacement (KO-SR, Invitrogen), 1 % non-essential amino acids (NEAA, Lonza), 1 % Glutamax (Invitrogen), 50 U/mL penicillin/streptomycin (Lonza), and recombinant human basic fibroblast growth factor (R&D Systems). The cells were passaged once a week by treating the pluripotent stem cell colonies with collagenase IV (Gibco) and seeding them onto a fresh MEF feeder layer.

### Cardiomyocyte differentiation

Cardiomyocytes were differentiated from hESCs as well as from the control and LQT1 and -2-specific hiPSCs using the mouse visceral endoderm-like (END-2) cell co-culture method, as described elsewhere (Mummery et al. 2003). Briefly, mitomycin C (Sigma) -inactivated END-2 cells were trypsinized and plated onto 12-well plates in 0 % KO-SR hES medium. The pluripotent stem cells were plated on top of the END-2 cell monolayer the following day. The 0 % KO-SR hES medium was refreshed on days 5, 8 and 12. At day 14, the medium was changed to 10 % KO-SR hES medium, and it was refreshed three times a week thereafter.

### Pharmacological testing

#### Drugs

Cisapride monohydrate, quinidine and (±)-sotalol hydrochloride were obtained from Sigma-Aldrich and dissolved in dimethyl sulfoxide (Sigma-Aldrich) at 10 mM. E-4031 (Alomone Labs) and erythromycin (Abboticin i.v., Amdipharm) were dissolved in sterile H_2_O at 1 and 50 mg/mL, respectively. Isoprenaline (Isuprel i.v., Hospira) was supplied in ready-to-use ampoules. The drug concentrations were chosen based on their therapeutic blood serum concentration range (Schulz and Schmoldt 2003). The following concentrations of cisapride monohydrate, quinidine, (±)-sotalol hydrochloride, and erythromycin were tested: half the concentration of the lower limit for the therapeutic blood serum concentration, the lower limit of the therapeutic blood serum concentration range, the average therapeutic serum concentration, the upper limit of the therapeutic range, and twice the upper limit of the therapeutic concentration (Schulz and Schmoldt 2003). The isoprenaline concentrations were chosen according to previously published values and multiples thereof (Pekkanen-Mattila et al. 2009). The drugs were serially diluted in embryoid body (EB)-medium with 5 % fetal bovine serum (FBS, see below). The test concentrations for each drug are listed in Table 2.

**Table 2:** Drugs and their concentrations used with cardiomyocytes derived from human embryonic and induced pluripotent stem cells

Drug	Concentrations
Isoprenaline	nM: 81, 402
Cisapride monohydrate	nM: 40, 83, 120, 165, 330
Erythromycin	µM: 1.5, 3, 5.5, 8, 16
Sotalol	µM: 0.8, 1.6, 5.7, 9.7, 19.4
Quinidine	µM: 1.5, 3, 9, 15, 30
E-4031	nM: 10, 100, 300, 500, 700

#### Multielectrode array recordings

The hESC-CMs and the control, LQT1-, and LQT2-specific hiPSC-CM aggregates were mechanically excised from the cultures and plated onto fetal bovine serum (PAA) and 0.1 % gelatin- (Sigma-Aldrich) coated 6-well-MEAs (6wellMEA200/30iR-TI-mr, MultiChannel Systems MCS GmbH). The FPs were recorded with a USB-MEA1060 amplifier, and the temperature was kept at +37 °C using a TC02 heating element (both from Multi Channel Systems MCS GmbH). The MEAs were covered with gas-permeable MEA membranes (ALA MEA-SHEET, ALA Scientific) during the recordings. A wash-in time of 2 min was allowed for the drugs before recordings. The beating areas were cultured in EB medium consisting of KO-DMEM (Invitrogen) supplemented with 20 % FBS (Invitrogen), 1 % NEAA (Lonza), 1 % Glutamax (Invitrogen), and 50 U/mL penicillin/streptomycin (Lonza). The drug tests were performed in 5 % FBS containing EB medium; the medium was changed at least an hour prior to the baseline recordings, and the cells were allowed to stabilize in the incubator (+37 °C, 5 % CO_2_). The field potential signals were recorded with MC_Rack v.4.0.0 software (Multi Channel Systems MCS GmbH).

### Data and statistical analysis

The field potential durations (FPDs) from the recorded files were measured offline using our in-house developed CardioMDA software (Pradhapan et al. 2013) and AxoScope10 (Molecular Devices). The baseline data are presented as mean ± standard deviation (SD). Linear mixed-effect models with either Bazett’s corrected field potential duration (cFPD) or beating rate (BR) as a dependent variable were fitted using the function lme in R (Software environment for statistical computing and graphics, version 2.13.0, The R Foundation for Statistical Computing). The factorial variable for the concentration level was used as an independent variable. The lowest concentration level was used as a reference group. Random intercept for different aggregates was used together with independent random errors.

## Results

### LQT patient characteristics

Skin biopsies were obtained from two siblings, a 28-year old female asymptomatic LQT1 patient (QTc interval, 428 ms) and a 41-year old female symptomatic LQT1 patient (QTc interval, 456 ms), both with the *KCNQ1* G589D mutation. In addition, skin biopsies were obtained from a 61-year old male asymptomatic LQT2 patient with the *KCNH2* R176W mutation (QTc interval, 437 ms). The symptomatic LQT1 patient had experienced seizures before beta-blocker medication, while the asymptomatic sibling has never had any cardiac symptoms. The asymptomatic LQT2 individual has only experienced occasional palpitations, which although are not specific symptoms of LQTS.

### Molecular characteristics of cardiomyocytes

Reverse-transcription PCR and immunofluorescence microscopy showed that the control and LQT-hiPSCs expressed the pluripotency markers NANOG, OCT3/4, SOX2, SSEA4, TRA-1-60, and TRA-1-81 (Additional file 1: Fig. S1A, Table S1). Furthermore, the hiPSC colony morphology resembled those of hESCs, the hiPSCs differentiated into cardiomyocytes at efficiencies previously reported for hESCs upon END-2 co-culture (data not shown) (Pekkanen-Mattila et al. 2009), showed nuclear localization of the cardiac-specific transcription factor NKX2.5 which is also expressed in hESC-CMs (Asp et al. 2010), and exhibited a distinct striated pattern in cardiac troponin T staining (Additional file 1: Fig. S1B). The electrophysiological properties and drug responses were recorded with microelectrode arrays (MEAs).

### Effects of cardioactive drugs to the cFPD of hiPSC-CMs

We compared the drug responses of the control cardiomyocytes derived from hESC line H7 (hereafter called hESC) and two wild-type hiPSC lines UTA.00112.hFF (hereafter called WTa) and UTA.01006.WT (hereafter called WTb) to the hiPSC-derived LQT-CMs (for the cell line properties, see Table 1). The results of the clonal cell lines derived from the same individual were pooled together (UTA.00208.LQT1 and UTA.00211.LQT1 [=LQT1A], UTA.00303.LQT1 and UTA.00313.LQT1 [=LQT1B], UTA.00514.LQT2 and UTA.00525.LQT2 [=LQT2]). LQT1A were derived from a symptomatic patient, LQT1B from an asymptomatic mutation carrier and LQT2 from asymptomatic mutation carrier.

#### Baseline characteristics

**Fig. 1:**
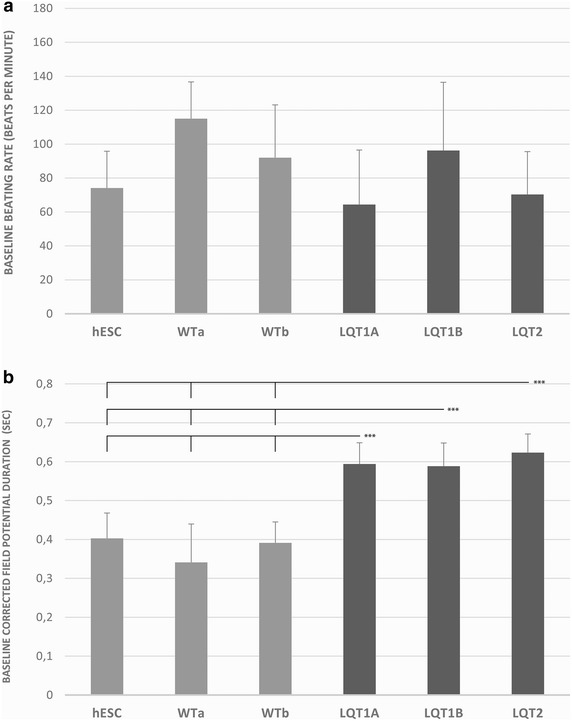
Baseline cardiac parameters. **a** The beating rate (BR) of the cardiomyocyte clusters differentiated from each human induced pluripotent stem cell (hiPSC) line. **b** The rate-corrected field potential duration (cFPD) for the same cardiomyocyte clusters. Significance level is indicated by ***p < 0.001

The baseline beating rates (BR) showed large differences between cell populations, ranging from 59 to 105 beats per minute (Fig. 1a). However, these BRs did not differ significantly among cardiomyocytes differentiated from hESCs, control-, or LQT-hiPSCs. In contrast, the beating rate-corrected field potential duration (cFPD, Bazett´s) showed significant differences between control- and LQT-CMs (Fig. 1b). At baseline level, the LQT-CMs showed significantly longer cFPD (586–600 ms) compared to control-CMs (340–400 ms) (p < 0.001).

#### Isoprenaline effects

**Fig. 2:**
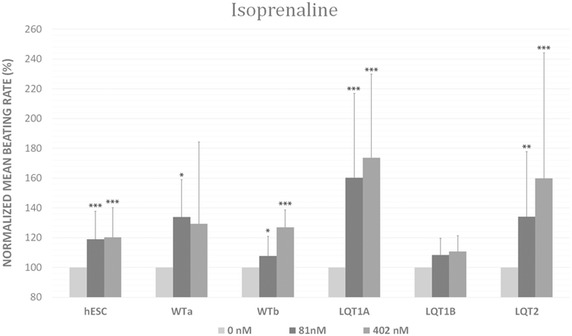
Isoprenaline response of the control and long QT syndrome (LQT) type 1 and 2-specific cardiomyocyte clusters (hESC, WTa, WTb, LQT1A, LQT1B and LQT2). Data is presented mean ± SD. Mean beating rates (BR) were normalized to baseline BR. *Asterisks mark* the statistical significance for mean BR increase compared to baseline values. *p < 0.05, **p < 0.01, ***p < 0.001

To test whether the differentiated cardiomyocytes had proper β-adrenergic responses, we subjected them to an isoprenaline challenge. This treatment had chronotropic effect on cardiomyocytes and increased the BR at the concentration of 81 and 402 nM (Fig. 2). Isoprenaline did not induce any arrhythmic beats in any of the CMs, including those derived from LQT1- and LQT2-hiPSC lines.

#### Effects of non-cardiac drugs cisapride and erythromycin

**Fig. 3:**
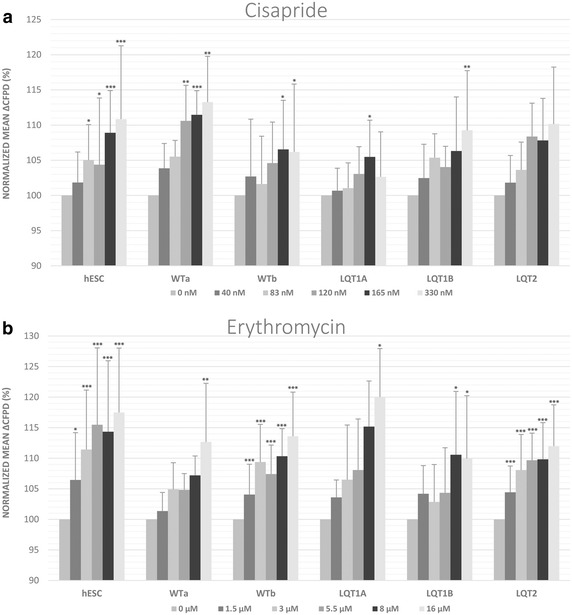
Non-cardiac drug responses of the control and long QT syndrome (LQT) type 1 and 2-specific cardiomyocyte clusters (hESC, WTa, WTb, LQT1A, LQT1B and LQT2). The drugs used were **a** cisapride and **b** erythromycin. Changes in the rate-corrected field potential duration (cFPD) were calculated as relative changes from the baseline for each cardiomyocyte cluster. Data is presented mean ± SD. Mean ΔcFPDs were normalized to baseline cFPD. *Asterisks mark* the statistical significance for mean ΔcFPD prolongation compared to baseline values. *p < 0.05, **p < 0.01, ***p < 0.001

Cisapride increased the repolarization time in all CMs studied, although this effect did not reach statistical significance in the LQT2-CMs, apparently due to large experimental variation (Fig. 3a).

Erythromycin likewise induced a significant average increase of cFPD in all cell lines studied, in a concentration range of 1.5–16 µM (Fig. 3b). No arrhythmic beats or beating arrests were observed with cisapride or with erythromycin.

#### Effects of the anti-arrhythmic drugs sotalol and quinidine

**Fig. 4:**
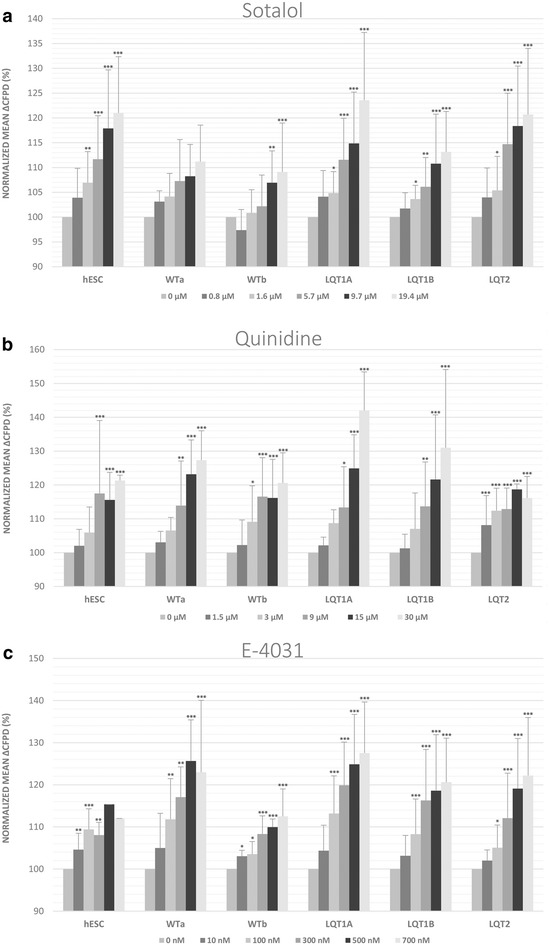
Class I and class III anti-arrhythmic drug responses of the control and long QT syndrome (LQT) type 1 and 2-specific cardiomyocyte clusters (hESC, WTa, WTb, LQT1A, LQT1B and LQT2). The drugs used were **a** sotalol, **b** quinidine and **c** E-4031. During E-4031 challenge all but one of the cardiac aggregates in hESC-CMs became arrhythmic or stopped beating at concentrations of 500–700 nM. Thus, *error bars* indicating standard deviation could not be calculated for these experimental points. Changes in the rate-corrected field potential duration (cFPD) were calculated as relative changes from the baseline for each cardiomyocyte cluster. Data is presented mean ± SD. Mean ΔcFPDs were normalized to baseline cFPD. *Asterisks mark* the statistical significance for mean ΔcFPD prolongation compared to baseline values. *p < 0.05, **p < 0.01, ***p < 0.001

Upon sotalol (0.8–19.4 µM) challenge, the maximal cFPD prolongation ranged from of 8 % (WTb-CMs) to 23 % (LQT1A-CMs) (Fig. 4a). Quinidine also caused a concentration-dependent (1.5–30 µM) average cFPD prolongation in all the cardiomyocytes (Fig. 4b). Of all clinically used drugs tested (cisapride, erythromycin, sotalol, quinidine), quinidine showed the largest cFPD-increasing effect (31–42 %) seen in LQT1-CMs.

#### The effect of the hERG channel blocker E-4031

E-4031 also caused concentration-dependent cFPD prolongation in all cardiomyocyte types (Fig. 4c). It is of note that in hESC-CMs all but one of the cardiac aggregates became arrhythmic or stopped beating at concentrations of 500–700 nM. Thus, error bars indicating standard deviation could not be calculated for these experimental points. The cFPD prolongation in the different cell lines reached statistical significance at E-4031 concentration of 100 nM indicating that the sensitivities of the different cell lines to E-4031 were very similar (Fig. 4c).

**Fig. 5:**
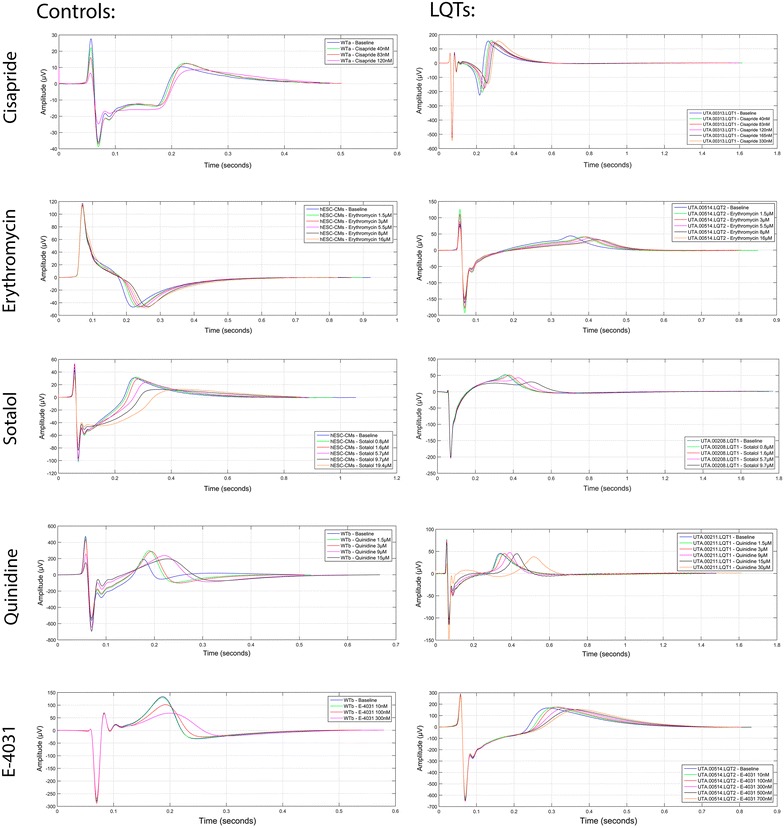
Representative traces of cisapride, erythromycin, sotalol, quinidine and E-4031 in control- and long QT-cardiomyocytes (LQT-CMs)

In summary, Fig. 5 illustrates the representative traces of cisapride, erythromycin, sotalol, quinidine and E-4031 in control- and LQT-CMs.

### The proarrhythmic effects of sotalol, quinidine and E-4031

Sotalol, quinidine and the hERG blocker E-4031 showed proarrhythmic effects on certain individual cell lines (Table 3). With sotalol, only spontaneous afterdepolarization (AD)-like waveform abnormalities (but not beating arrests) were observed (Table 3). Sotalol caused AD-like waveforms ranging from concentrations 5.7 µM (average therapeutic serum concentration) to 19.4 µM (twice the upper therapeutical serum concentration). None of the LQT1B-CMs showed any arrhythmic behavior upon sotalol challenge.

**Table 3:** Arrhythmic effects of sotalol, quinidine, and E-4031 observed during the field potential (FP) recordings

Sotalol (µM)	hESC (n = 11)	WTa (n = 6)	WTb (n = 12)	LQT1A (n = 13)*^,s^	LQT1B (n = 13)*^,a^	LQT2 (n = 15)^a^
0.8						
1.6						
5.7						AD(2)
9.7	AD(4)		AD(1)			
19.4		AD(1)		AD(2)		

Quinidine (µM)	hESC (n = 10)	WTa (n = 6)	WTb (n = 11)	LQT1A (n = 12)*^,s^	LQT1B (n = 16)*^,a^	LQT2 (n = 12)^a^
1.5						
3						AD(1)
9	AD(3)		BA(7)	AD(1)	BA(5)	BA(5)
15		BA(2)		BA(2)		
30	BA(1)					

E-4031 (nM)	hESC (n = 10)	WTa (n = 6)	WTb (n = 8)	LQT1A (n = 16)*^,s^	LQT1B (n = 16)*^,a^	LQT2 (n = 13)^a^
10						
100						BA(4)
300	AD(7)	AD(2)		AD(8)		AD(3)
500	BA(1)	BA(2)	BA(1)		AD(4)	
700					BA(1)	

The reported values in the table are the lowest concentrations that induced the effect. The numbers in parentheses indicate how many cardiomyocyte clusters in total were affected

*AD* afterdepolarization (AD)-waveforms, *BA* beating arrest (termination of FP activity)

* Siblings ^s^ symptomatic ^a^ asymptomatic

Quinidine showed also beating arrests in addition to AD-like waveform abnormalities (Table 3). The AD-like waveforms were observed at the therapeutical serum concentration range (3–15 µM) in hESC-, LQT1A- and LQT2-CMs but not in WT- or LQT1B-CMs (Table 3). On the other hand, the beating arrests occurred ranging from 9 µM (average therapeutic serum concentration) to 30 µM (twice the upper therapeutical serum concentration) (Table 3).

The compound E-4031 also caused AD-like waveform abnormalities and beating arrests (Table 3). E-4031 caused AD-like waveform abnormalities in cardiomyocytes differentiated from all of the lines except WTb-CMs. Furthermore, beating arrests were seen in all cardiomyocyte types except LQT1A-CMs. Among the LQTs, the highest frequency for AD-like waveform abnormalities was found in LQT1A-CMs (50 % of cardiomyocyte aggregates). On the other hand, the highest frequency for beating arrests were seen in LQT2-CMs (31 % of cardiomyocyte aggregates).

## Discussion

The present study further demonstrates the usefulness of the hiPSC-derived cardiomyocytes to serve as a model for long QT syndrome, showing that the QT interval-prolonging effects of clinically used drugs are replicated in this in vitro system, using concentrations that are within or close to their therapeutic range (Schulz and Schmoldt 2003). We showed that LQT-CMs corrected field potential duration is significantly longer than in control-CMs at baseline conditions, which suggests that long QT syndrome is recapitulated in these cells. This study also reproduces in cell clusters the earlier observation with single cells by patch clamp with prolonged APDs in LQT-CMs compared to controls (Kiviaho et al. 2015; Lahti et al. 2012). In the current study LQT- and control CMs were further exposed to various cardioactive drugs to investigate their effects.

The cardiomyocytes beating rates were increased by isoprenaline, which is a β-adrenergic agonist that affects calcium (I_Ca,L_) channel activation via β_1_ receptors. This is in line with the previous studies showing positive chronotropic effect of isoprenaline on both hESC- and hiPSC-CMs (Moretti et al. 2010; Pekkanen-Mattila et al. 2009; Yokoo et al. 2009). β-adrenergic stimulation with isoprenaline did not have any arrhythmic effects on the LQT1 lines although LQT1 patients often experience symptoms at increased heart rates.

Cisapride is a serotonin 5-HT4 receptor agonist that has been reported to block the hERG potassium channel (Mohammad et al. 1997). It is known to induce TdP in patients (Darpö 2001). Caspi et al. (2009) reported significant (>50 nM) dose-dependent prolongation of cFPD in hESC-CMs (Braam et al. 2010; Caspi et al. 2009). We obtained similar results as Caspi et al. (2009) as the hESC-CMs used in our study showed dose-dependent and significant cFPD prolongation starting at 83 nM. In contrast, Braam et al. (2010) reported no significant cisapride-induced cFPD prolongation in hESC-CMs. However, they judged the therapeutic concentration to be 2.6–4.9 nM (Redfern et al. 2003), whereas we chose the therapeutic range as 83–165 nM (Schulz and Schmoldt 2003), which may explain the fact that they were not able to detect significant cisapride-induced cFPD prolongation. Mehta et al. (2013) have also investigated the effect of cisapride to the cFPD of 150–170 days old EB-based hiPSC-derived CMs. Likewise, they also found concentration-dependent cFPD prolongation (Mehta et al. 2013). Mehta et al. (2013) did not observe any arrhythmias with cisapride, which is in line with our study here.

Erythromycin is a macrolide antibiotic that prolongs the QT interval in patients (Shaffer et al. 2002). The therapeutic plasma concentration of erythromycin has been reported to be 3–8 µM (Schulz and Schmoldt 2003). In our study, erythromycin prolonged significantly cFPD in hESC-CMs already at the first test concentration (1.5 µM). Our data may suggest a slightly higher sensitivity of LQT2-CMs compared to LQT1-CMs, but high experimental variation precludes firm conclusions. Erythromycin did not induce any arrhythmic effects in the cardiomyocytes.

Sotalol is a β-adrenergic receptor antagonist and a Vaughan–Williams class III anti-arrhythmic agent that potently blocks the potassium ion channels in cardiomyocytes (Edvardsson et al. 1980). In hESC-CMs, Braam et al. (2010) reported cFPD prolongations of 15–20 % at sotalol concentrations of 1.8–14 µM (Braam et al. 2010). In a subsequent study, they also observed 20 % cFPD prolongation in hESC-CMs at sotalol concentration of 10 µM (Braam et al. 2013). Our study resulted in similar data. At concentrations of 6–10 µM, we observed a cFPD prolongation of 12–18 % in the hESC-CMs, increasing to 21 % at 19 µM. We observed that sotalol increased cFPD in all the CMs at the therapeutical concentration range (1.6–9.7 µM). In line with our data, Mehta et al. (2013) reported similar observations from sotalol-induced cFPD prolongation in hiPSC-derived CMs (Mehta et al. 2013). Furthermore, according to our data sotalol significantly increased cFPD at earlier concentrations in LQT-CMs than in WTa or WTb. This may suggest that LQT-CMs may be more vulnerable to the cFPD-prolonging effect of sotalol than hiPSC-derived control cells.

Quinidine is a class Ia antiarrhythmic drug that primarily blocks sodium ion channels at high concentrations and hERG channel at low concentrations and is associated with TdP (Bauman et al. 1984). Quinidine prolonged the cFPDs in all of our cell lines ranging from 16 to 42 %. Braam et al. (2010) reported a prolongation of 20–50 % in hESC-CMs with quinidine concentrations of up to 3.2 µM and increases in the cFPD at higher concentrations, and Caspi et al. (2009) reported a cFPD prolongation of slightly <30 % in hESC-CMs with 8 µM quinidine (Braam et al. 2010; Caspi et al. 2009). Mehta et al. (2013) reported a 20 and 55 % cFPD prolongation in 150–170 days old hiPSC-CMs at 1 and 10 µM concentrations, respectively. Here, we noticed an 18 % increase in the cFPD of the hESC-CMs at a concentration of 9 µM. Quinidine induced beating arrests which emerged at the average or upper therapeutic serum concentration. Quinidine is known to inhibit sodium ion channels at high concentrations (Snyders and Hondeghem 1990), which may be the reason for higher frequency of beating arrest than AD-like waveform abnormalities. The LQT2-CMs, showed significantly prolonged cFPD already at the half of the lower limit of therapeutic concentration (1.5 µM), unlike control- or LQT1-CMs, and AD-like waveform abnormalities at the lower limit of therapeutic concentration level (3 µM). These findings together may imply that LQT2 harboring hERG R176 W may be at more increased risk for quinidine-induced cFPD prolongation and proarrhythmic effects than control- or LQT1-CMs harboring G589D mutation. However, we emphasize that our findings, based on cell lines derived from three LQTS patients only, must be interpreted with caution.

E-4031 is a hERG channel blocker (Caspi et al. 2009) that reduced the I_Kr_ current during cardiac repolarization and caused prolongations in all our cell lines. Braam et al. (2010) reported FPD prolongation in hESC-CMs treated with 30 nM E-4031 and AD-like waveform abnormalities at concentrations of 1 µM and over (Braam et al. 2010). In hiPSC-CM clusters similar cFPD prolongations have been reported as well (Mehta et al. 2011, 2014). We observed that the cFPD increased significantly more in CMs derived from symptomatic LQT1 patient than from asymptomatic. Presently, it is not known whether this indicates that symptomatic LQT1 may be at more increased risk for hERG blocker-induced cFPD increase. Egashira et al. (2012) reported cFPD prolongation of 20 % in LQT1-CMs derived from a symptomatic patient, when treated with 100 nM E-4031. These LQT1-CMs had a heterozygous deletion mutation in *KCNQ1*, 1893delC (Egashira et al. 2012). Here, we found cFPD prolongation of 13 and 8 % in LQT1A- and LQT1B-CMs carrying a G589D missense mutation. Egashira et al. (2012) also reported AD-like waveform abnormalities in LQT1-CMs treated with 300 nM E-4031, which is in line with our results from symptomatic LQT1A-CMs.

Variations of the in vitro responses to QTc interval affecting drugs may result from several reasons. Differences in the common polymorphisms regulating cardiac repolarization may result in the modulation of drug responses (Roden 2004). Furthermore, differences in drug responses between the LQT1 and -2 subtypes may result from differences in their respective repolarization reserves (Roden 1998). Other possible explanations may come from the observations that hPSC-CMs can have very low or very high spontaneous beating rates, and that the hPSC-CMs with BR ≥ 50 bpm express more atrial-related genes and therefore might be more atrial-like (Asp et al. 2010). Different cardiomyocyte subtypes (e.g., atrial vs. ventricular) may have different ion channel compositions (Gaborit et al. 2007). Therefore, for certain applications, if the beating rates are representative of differences between cardiac subtypes, then the drug responses might vary accordingly. We did not test whether the END-2 differentiation protocol used in this study generated more nodal-, atrial- or ventricular-like CMs across the cell lines. The MEA analysis used in the present study includes only beating clusters, suggesting the presence of nodal-, atrial- and ventricular-like type of CMs. However, previous study has shown that END-2 differentiation protocol generates mostly ventricular CMs shown by electrophysiological measurements (Mummery et al. 2003). It is also unknown whether the heterozygous mutated ion channels are assembled similarly in all differentiating cardiomyocytes. Variation in the ion channel composition from cluster to cluster and even from cell to cell might lead to differential drug responses. There are some evidence that there may be differences in same certain drug responses between clusters of CMs and monolayer CMs. In disease-free hiPSC-CM monolayers, Harris et al. (2013) reported statistically significant E-4031 induced cFPD prolongation already at 3 nM whereas for disease-free clusters of hESC- or hiPSC-CMs the significant cFPD prolongations were reported to be found in 30–300 nM (Braam et al. 2010; Caspi et al. 2009; Mehta et al. 2011, 2014). In disease-free hiPSC-CM monolayers E-4031 evoked arrhythmias were seen as early as 10–30 nM (Harris et al. 2013; Nakamura et al. 2014). We have also observed similar phenomenon in monolayer-based disease-free CMs with E-4031 (data not published). The aforementioned differences may have significant implications in drug screenings and would require further investigation.

### Potential limitation of the study

The clinical phenotypes of the patients did not correlate with repolarization times in this study. The occurrence of clinical symptoms in LQT-patients harboring G589D have been linked to QTc value. In the group of QTc > 500 ms over 50 % of the patients were symptomatic whereas only 16 % were symptomatic with QTc < 440 ms (Piippo et al. 2001). In addition, patients with C-terminal mutation such as G589D have milder phenotypes compared to transmembrane domain mutations of the same channel (Donger et al. 1997; Larsen et al. 1999; Piippo et al. 2001; Swan et al. 1999). In this study, the LQT1-G589D-patients’ QTc values were 456 and 428 ms for symptomatic and asymptomatic, respectively. In LQT2, R176W mutation has been reported elsewhere as a relatively mild form with modest QTc prolongation (Fodstad et al. 2006; Marjamaa et al. 2009). Here, the LQT2 patient had been asymptomatic except for occasional palpitations. In the present study, at the baseline significant differences in repolarization times were observed, but the drug effects appeared to be of similar magnitude in LQT-CMs harboring G589D or R176W mutations compared to healthy controls at this sample size. Given the large variance in cFPD it may be possible that by increasing the sample size statistical significances could be found between control and LQT-CMs. However, it should be emphasized that increases in repolarization times which are already at baseline longer are potentially more severe that in those of normal length.

## Conclusion

In conclusion, the present study reports in vitro electrophysiological effects of various non-cardiac and cardioactive drugs as assessed in hiPSC-derived LQT-cardiomyocytes. We have shown that long QT syndrome is recapitulated in hiPSC-derived LQT-cardiomyocytes. All drugs tested showed a systematic tendency to prolong the field-potential duration (cFPD, an indicator of repolarization phase). We did not observe any systematic differences in the sensitivities of cFPD prolongation among different drugs between control- and LQT-cardiomyocytes. Further studies are needed to explore the possibility that this system could aid in the patient-specific risk assessment when considering the use of potentially QT-interval prolonging drugs.

## Additional file

10.1186/s40064-016-1889-y Immunocytochemical marker expression of human induced pluripotent stem cells (hiPSCs) and their cardiomyocyte derivatives. **Table S1.** Pluripotency marker expression of the hPSCs. (n.d.: not determined).

## References

[CR1] Asp J, Steel D, Jonsson M, Ameen C, Dahlenborg K, Jeppsson A, Lindahl A, Sartipy P (2010). Cardiomyocyte clusters derived from human embryonic stem cells share similarities with human heart tissue. J Mol Cell Biol.

[CR2] Bauman JL, Bauernfeind RA, Hoff JV, Strasberg B, Swiryn S, Rosen KM (1984). Torsade de pointes due to quinidine: observations in 31 patients. Am Heart J.

[CR3] Bellin M, Casini S, Davis RP, D’Aniello C, Haas J, Ward-van Oostwaard D, Tertoolen LG, Jung CB, Elliott DA, Welling A (2013). Isogenic human pluripotent stem cell pairs reveal the role of a KCNH2 mutation in long-QT syndrome. EMBO J.

[CR4] Braam SR, Tertoolen L, van de Stolpe A, Meyer T, Passier R, Mummery CL (2010). Prediction of drug-induced cardiotoxicity using human embryonic stem cell-derived cardiomyocytes. Stem Cell Res.

[CR5] Braam SR, Tertoolen L, Casini S, Matsa E, Lu HR, Teisman A, Passier R, Denning C, Gallacher DJ, Towart R (2013). Repolarization reserve determines drug responses in human pluripotent stem cell derived cardiomyocytes. Stem Cell Res.

[CR6] Caspi O, Itzhaki I, Kehat I, Gepstein A, Arbel G, Huber I, Satin J, Gepstein L (2009). In vitro electrophysiological drug testing using human embryonic stem cell derived cardiomyocytes. Stem Cells Dev.

[CR7] Darpö B (2001). Spectrum of drugs prolonging QT interval and the incidence of torsades de pointes. Eur Heart J Suppl.

[CR8] Donger C, Denjoy I, Berthet M, Neyroud N, Cruaud C, Bennaceur M, Chivoret G, Schwartz K, Coumel P, Guicheney P (1997). KVLQT1 C-terminal missense mutation causes a forme fruste long-QT syndrome. Circulation.

[CR9] Edvardsson N, Hirsch I, Emanuelsson H, Ponten J, Olsson SB (1980). Sotalol-induced delayed ventricular repolarization in man. Eur Heart J.

[CR10] Egashira T, Yuasa S, Suzuki T, Aizawa Y, Yamakawa H, Matsuhashi T, Ohno Y, Tohyama S, Okata S, Seki T (2012). Disease characterization using LQTS-specific induced pluripotent stem cells. Cardiovasc Res.

[CR11] Fodstad H, Swan H, Laitinen P, Piippo K, Paavonen K, Viitasalo M, Toivonen L, Kontula K (2004). Four potassium channel mutations account for 73 % of the genetic spectrum underlying long-QT syndrome (LQTS) and provide evidence for a strong founder effect in finland. Ann Med.

[CR12] Fodstad H, Bendahhou S, Rougier JS, Laitinen-Forsblom PJ, Barhanin J, Abriel H, Schild L, Kontula K, Swan H (2006). Molecular characterization of two founder mutations causing long QT syndrome and identification of compound heterozygous patients. Ann Med.

[CR13] Gaborit N, Le Bouter S, Szuts V, Varro A, Escande D, Nattel S, Demolombe S (2007). Regional and tissue specific transcript signatures of ion channel genes in the non-diseased human heart. J Physiol.

[CR14] Harris K, Aylott M, Cui Y, Louttit JB, McMahon NC, Sridhar A (2013). Comparison of electrophysiological data from human-induced pluripotent stem cell-derived cardiomyocytes to functional preclinical safety assays. Toxicol Sci.

[CR15] Itzhaki I, Maizels L, Huber I, Zwi-Dantsis L, Caspi O, Winterstern A, Feldman O, Gepstein A, Arbel G, Hammerman H (2011). Modelling the long QT syndrome with induced pluripotent stem cells. Nature.

[CR16] Kiviaho AL, Ahola A, Larsson K, Penttinen K, Swan H, Pekkanen-Mattila M, Venäläinen H, Paavola K, Hyttinen J, Aalto-Setälä K (2015). Distinct electrophysiological and mechanical beating phenotypes of long QT syndrome type 1-specific cardiomyocytes carrying different mutations. IJC Heart Vasc.

[CR17] Lahti AL, Kujala VJ, Chapman H, Koivisto AP, Pekkanen-Mattila M, Kerkela E, Hyttinen J, Kontula K, Swan H, Conklin BR (2012). Model for long QT syndrome type 2 using human iPS cells demonstrates arrhythmogenic characteristics in cell culture. Dis Models Mech.

[CR18] Larsen LA, Fosdal I, Andersen PS, Kanters JK, Vuust J, Wettrell G, Christiansen M (1999). Recessive romano-ward syndrome associated with compound heterozygosity for two mutations in the KVLQT1 gene. Eur J Hum Genet.

[CR19] Marjamaa A, Salomaa V, Newton-Cheh C, Porthan K, Reunanen A, Karanko H, Jula A, Lahermo P, Vaananen H, Toivonen L (2009). High prevalence of four long QT syndrome founder mutations in the finnish population. Ann Med.

[CR20] Matsa E, Rajamohan D, Dick E, Young L, Mellor I, Staniforth A, Denning C (2011). Drug evaluation in cardiomyocytes derived from human induced pluripotent stem cells carrying a long QT syndrome type 2 mutation. Eur Heart J.

[CR21] Matsa E, Dixon JE, Medway C, Georgiou O, Patel MJ, Morgan K, Kemp PJ, Staniforth A, Mellor I, Denning C (2014). Allele-specific RNA interference rescues the long-QT syndrome phenotype in human-induced pluripotency stem cell cardiomyocytes. Eur Heart J.

[CR22] Mehta A, Chung YY, Ng A, Iskandar F, Atan S, Wei H, Dusting G, Sun W, Wong P, Shim W (2011). Pharmacological response of human cardiomyocytes derived from virus-free induced pluripotent stem cells. Cardiovasc Res.

[CR23] Mehta A, Chung Y, Sequiera GL, Wong P, Liew R, Shim W (2013). Pharmacoelectrophysiology of viral-free induced pluripotent stem cell-derived human cardiomyocytes. Toxicol Sci.

[CR24] Mehta A, Verma V, Nandihalli M, Ramachandra CJ, Sequiera GL, Sudibyo Y, Chung Y, Sun W, Shim W (2014). A systemic evaluation of cardiac differentiation from mRNA reprogrammed human induced pluripotent stem cells. PLoS One.

[CR25] Mohammad S, Zhou Z, Gong Q, January CT (1997). Blockage of the HERG human cardiac K+ channel by the gastrointestinal prokinetic agent cisapride. Am J Physiol.

[CR26] Moretti A, Bellin M, Welling A, Jung CB, Lam JT, Bott-Flugel L, Dorn T, Goedel A, Hohnke C, Hofmann F (2010). Patient-specific induced pluripotent stem-cell models for long-QT syndrome. N Engl J Med.

[CR27] Mummery C, Ward-van Oostwaard D, Doevendans P, Spijker R, van den Brink S, Hassink R, van der Heyden M, Opthof T, Pera M, de la Riviere AB (2003). Differentiation of human embryonic stem cells to cardiomyocytes: role of coculture with visceral endoderm-like cells. Circulation.

[CR28] Nakamura Y, Matsuo J, Miyamoto N, Ojima A, Ando K, Kanda Y, Sawada K, Sugiyama A, Sekino Y (2014). Assessment of testing methods for drug-induced repolarization delay and arrhythmias in an iPS cell-derived cardiomyocyte sheet: multi-site validation study. J Pharmacol Sci.

[CR29] Navarrete EG, Liang P, Lan F, Sanchez-Freire V, Simmons C, Gong T, Sharma A, Burridge PW, Patlolla B, Lee AS (2013). Screening drug-induced arrhythmia [corrected] using human induced pluripotent stem cell-derived cardiomyocytes and low-impedance microelectrode arrays. Circulation.

[CR30] Pekkanen-Mattila M, Kerkela E, Tanskanen JM, Pietila M, Pelto-Huikko M, Hyttinen J, Skottman H, Suuronen R, Aalto-Setala K (2009). Substantial variation in the cardiac differentiation of human embryonic stem cell lines derived and propagated under the same conditions: a comparison of multiple cell lines. Ann Med.

[CR31] Piippo K, Swan H, Pasternack M, Chapman H, Paavonen K, Viitasalo M, Toivonen L, Kontula K (2001). A founder mutation of the potassium channel KCNQ1 in long QT syndrome: implications for estimation of disease prevalence and molecular diagnostics. J Am Coll Cardiol.

[CR32] Pradhapan P, Kuusela J, Viik J, Aalto-Setala K, Hyttinen J (2013). Cardiomyocyte MEA data analysis (CardioMDA)—a novel field potential data analysis software for pluripotent stem cell derived cardiomyocytes. PLoS One.

[CR33] Redfern WS, Carlsson L, Davis AS, Lynch WG, MacKenzie I, Palethorpe S, Siegl PK, Strang I, Sullivan AT, Wallis R (2003). Relationships between preclinical cardiac electrophysiology, clinical QT interval prolongation and torsade de pointes for a broad range of drugs: evidence for a provisional safety margin in drug development. Cardiovasc Res.

[CR34] Roden DM (1998). Taking the “idio” out of “idiosyncratic”: predicting torsades de pointes. Pacing Clin Electrophysiol.

[CR35] Roden DM (2004). Drug-induced prolongation of the QT interval. N Engl J Med.

[CR36] Sanguinetti MC, Jiang C, Curran ME, Keating MT (1995). A mechanistic link between an inherited and an acquired cardiac arrhythmia: HERG encodes the IKr potassium channel. Cell.

[CR37] Schulz M, Schmoldt A (2003). Therapeutic and toxic blood concentrations of more than 800 drugs and other xenobiotics. Pharmazie.

[CR38] Schwartz PJ, Ackerman MJ, George AL, Wilde AA (2013). Impact of genetics on the clinical management of channelopathies. J Am Coll Cardiol.

[CR39] Shaffer D, Singer S, Korvick J, Honig P (2002). Concomitant risk factors in reports of torsades de pointes associated with macrolide use: review of the united states food and drug administration adverse event reporting system. Clin Infect Dis.

[CR40] Snyders DJ, Hondeghem LM (1990). Effects of quinidine on the sodium current of guinea pig ventricular myocytes. Evidence for a drug-associated rested state with altered kinetics. Circ Res.

[CR41] Swan H, Viitasalo M, Piippo K, Laitinen P, Kontula K, Toivonen L (1999). Sinus node function and ventricular repolarization during exercise stress test in long QT syndrome patients with KvLQT1 and HERG potassium channel defects. J Am Coll Cardiol.

[CR42] Takahashi K, Tanabe K, Ohnuki M, Narita M, Ichisaka T, Tomoda K, Yamanaka S (2007). Induction of pluripotent stem cells from adult human fibroblasts by defined factors. Cell.

[CR43] Wang Q, Curran ME, Splawski I, Burn TC, Millholland JM, VanRaay TJ, Shen J, Timothy KW, Vincent GM, de Jager T (1996). Positional cloning of a novel potassium channel gene: KVLQT1 mutations cause cardiac arrhythmias. Nat Genet.

[CR44] Wu SM, Hochedlinger K (2011). Harnessing the potential of induced pluripotent stem cells for regenerative medicine. Nat Cell Biol.

[CR45] Yokoo N, Baba S, Kaichi S, Niwa A, Mima T, Doi H, Yamanaka S, Nakahata T, Heike T (2009). The effects of cardioactive drugs on cardiomyocytes derived from human induced pluripotent stem cells. Biochem Biophys Res Commun.

[CR46] Yu J, Vodyanik MA, Smuga-Otto K, Antosiewicz-Bourget J, Frane JL, Tian S, Nie J, Jonsdottir GA, Ruotti V, Stewart R (2007). Induced pluripotent stem cell lines derived from human somatic cells. Science.

