# Supplementary material for: Effects of cardioactive drugs on human induced pluripotent stem cell derived long QT syndrome cardiomyocytes
Source: Springerplus. 2016 Feb 29;5:234. doi: 10.1186/s40064-016-1889-y (PMC4771667; doi:10.1186/s40064-016-1889-y)
Supplement: Supplementary file 1 — 10.1186/s40064-016-1889-y Immunocytochemical marker expression of human induced pluripotent stem cells (hiPSCs) and their cardiomyocyte derivatives. Table S1. Pluripotency marker expression of the hPSCs. (n.d.: not determined). [file 40064_2016_1889_MOESM1_ESM.doc]

# Additional file 1

## Additional file 1: Material and methods

### 1.1. Reprogramming of human induced pluripotent stem cells

Primary fibroblasts from skin biopsies were cultured in Dulbeco’s Modified Eagle Medium (DMEM, Lonza) supplemented with 10% fetal bovine serum (FBS, Lonza), 2 mM L-glutamine, and 50 U/mL penicillin/streptomycin. 293FT cells (Invitrogen) were cultured with 1% non-essential amino acids added in the medium (NEAA, Cambrex). PLAT-E-cells (Cell Biolabs), irradiated SNL-76/7 (HPA Culture Collections) and mouse embryonic fibroblasts (MEFs, Millipore) cells were cultured without antibiotics. Human iPSCs were maintained in KSR-medium consisting of knockout (KO)-DMEM (Invitrogen), 20% KO-serum replacement (KO-SR, Invitrogen), NEAA, L-glutamine, penicillin/streptomycin, 0.1 mM 2-mercaptoethanol, and 4 ng/mL basic fibroblast growth factor (bFGF, R&D Systems).

The control and patient-specific hiPSC lines were reprogrammed from the primary fibroblasts using lentiviral transduction and subsequent retroviral transduction as described before (Takahashi et al., 2007). The cells, plasmids and reagents used were as follows: 293FT-cells, PLAT-E-cells, pLenti6/UbC/mSlc7a1-vector (Addgene, ViraPower^TM^ Packaging Mix (Invitrogen), Lipofectamine^TM^2000 (Invitrogen), pMX retroviral vector (hOCT3/4, hSOX2, hKLF4 or hc-MYC, Addgene) and Fugene 6 (Roche Diagnostics).

### 1.2. Pluripotency marker expression

Passage 8 hiPSCs were fixed with 4% paraformaldehyde (Sigma-Aldrich) and stained with anti-OCT3/4 (1:400, R&D Systems), anti-TRA1-60 (1:200, Millipore), and anti-TRA1-81, anti-SOX2, anti-Nanog, and anti-SSEA4 (all 1:200, from Santa Cruz Biotechnology). Secondary antibodies were Alexa-Fluor-568-donkey-anti-goat-IgG, Alexa-Fluor-568-goat-anti-mouse-IgM or Alexa-Fluor-568-donkey-anti-mouse-IgG (all from Invitrogen).

### 1.3. Reverse-transcription polymerase chain reaction

For RT-PCR, mRNA was collected from hiPSC lines at passages 3, 6, and 11. H7 hESC mRNA was used as positive control and embryoid body (EB) mRNA as negative control. mRNA was purified using NucleoSpin RNA II -kit (Macherey-Nagel, Düren, Germany) and converted into cDNA using high-capacity cDNA RT -kit (Applied Biosystems, Carlsbad, CA, USA) according to manufacturers instructions. PCR was done with Dynazyme II (Finnzymes) using 1 µl cDNA as template and 2 µM primers. Transfected plasmids (hOCT3/4, hSOX2, hKLF4, and hc-MYC) were used as templates for positive controls of exogenous primers. PCR primers for hiPSC characterization and detailed reaction conditions have been described previously (Takahashi et al., 2007).

# References

Takahashi, K., Tanabe, K., Ohnuki, M., Narita, M., Ichisaka, T., Tomoda, K., and Yamanaka, S. (2007). Induction of Pluripotent Stem Cells from Adult Human Fibroblasts by Defined Factors. Cell *131*, 861-872.

## Additional file 1: Figures


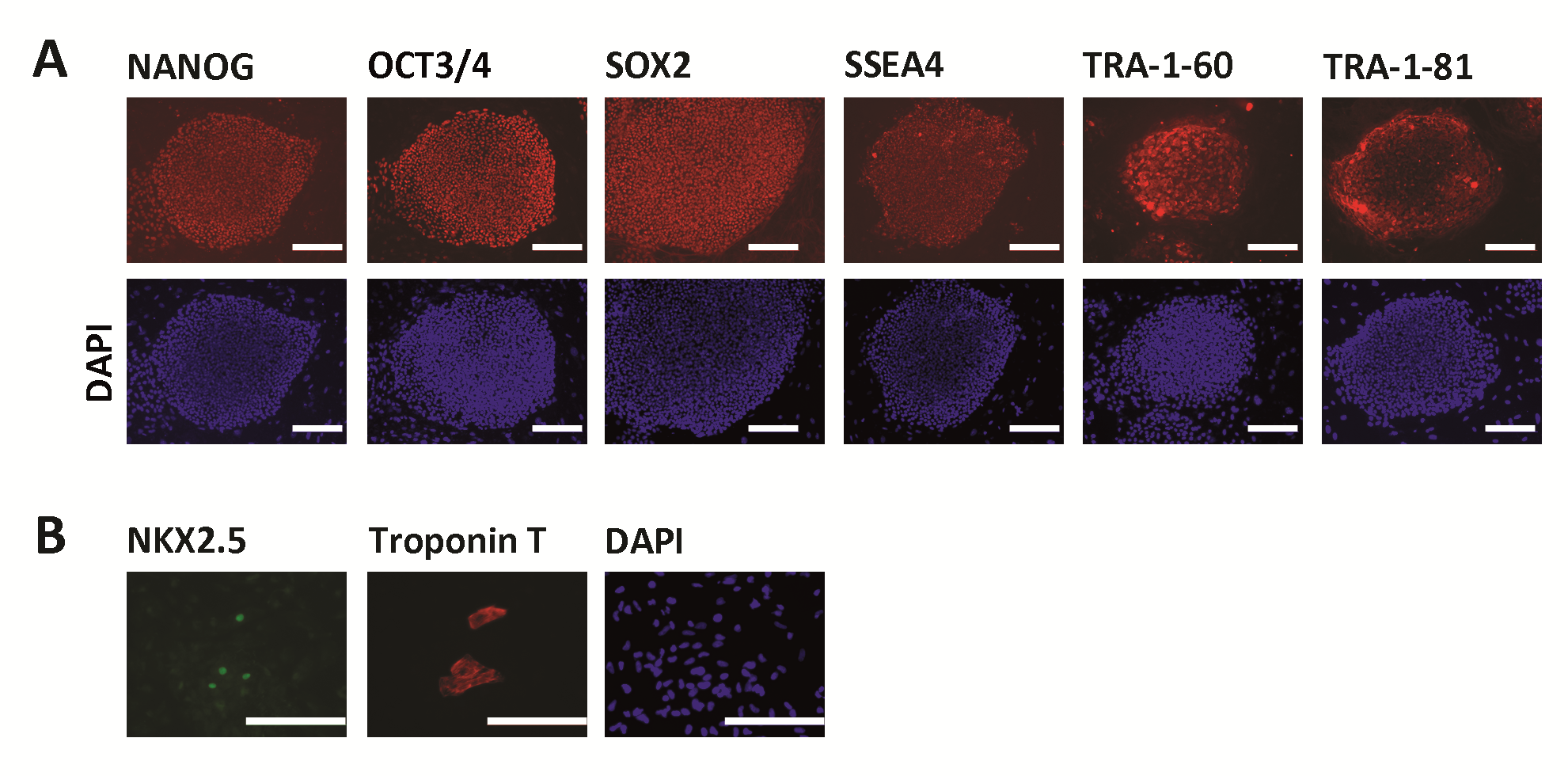


**Figure S1**. Immunocytochemical marker expression of human induced pluripotent stem cells (hiPSCs) and their cardiomyocyte derivatives. A) The hiPSC colonies express proper pluripotency markers (in red, corresponding nuclear staining with DAPI shown below in blue). B) The hiPSC-derived cardiomyocytes express the cardiac transcription factor NKX2.5 (green, located in the nucleus) and structural protein cardiac troponin T (red) (corresponding DAPI staining shown in blue, rightmost). Scale bars 200 µm.

## Additional file 1 : Tables

## **Table S1.** Pluripotency marker expression of the hPSCs. (n.d.:not determined).

| **Cell line** | **Karyotype** | **mRNA expression (RT-PCR)** | **Immunofluorescence** |
| --- | --- | --- | --- |
| hESC (H7) | 46XX | Endogenous:  *OCT3/4^+^, SOX2^+^, C-MYC^+^, REX1^+^, NANOG^+^*  Transgene:  *OCT3/4^-^, SOX2^-^, KLF4^-^, C-MYC^-^* | OCT3/4^+^  NANOG^+^  SSEA4^+^  TRA-1-60^+^ |
| UTA.00112.hFF (WTa) | 46XY | Endogenous:  *OCT3/4^+^, SOX2^+^, C-MYC^+^, REX1^+^, NANOG^+^*  Transgene:  *OCT3/4^-^, SOX2^-^, KLF4^-^, C-MYC^-^* | OCT3/4^+^  NANOG^+^  SSEA4 n.d.  TRA-1-60 n.d. |
| UTA.01006.WT  (WTb) | 46XX | Endogenous:  *OCT3/4^+^, SOX2^+^, C-MYC^-^, REX1^+^, NANOG ^+^*  Transgene:  *OCT3/4^-^, SOX2^-^, KLF4^-^, C-MYC^-^* | OCT3/4^+^  NANOG^+^  SSEA4^+^  TRA-1-60^+^ |
| UTA.00208.LQT1 | 46XX | Endogenous:  *OCT3/4^+^, SOX2^+^, C-MYC^-^, REX1^+^, NANOG ^+^*  Transgene:  *OCT3/4^-^, SOX2^-^, KLF4^-^, C-MYC^-^* | OCT3/4^+^  NANOG^+^  SSEA4^+^  TRA-1-60^+^ |
| UTA.00211.LQT1 | 46XX | Endogenous:  *OCT3/4^+^, SOX2^+^, C-MYC^-^, REX1^+^, NANOG ^+^*  Transgene:  *OCT3/4^-^, SOX2^-^, KLF4^-^, C-MYC^-^* | OCT3/4^+^  NANOG^+^  SSEA4^+^  TRA-1-60^+^ |
| UTA.00303.LQT1 | 46XX | Endogenous:  *OCT3/4^+^, SOX2^+^, C-MYC^+^, REX1^+^, NANOG ^+^*  Transgene:  *OCT3/4^-^, SOX2^-^, KLF4^-^, C-MYC^-^* | OCT3/4^+^  NANOG^+^  SSEA4^+^  TRA-1-60^+^ |
| UTA.00313.LQT1 | 46XX | Endogenous:  *OCT3/4* n.d.*, SOX2^+^, C-MYC* n.d.*, REX1* n.d.*, NANOG ^+^*  Transgene:  *OCT3/4^-^, SOX2^-^, KLF4^-^, C-MYC^-^* | OCT3/4^+^  NANOG^+^  SSEA4^+^  TRA-1-60^+^ |
| UTA.00514.LQT2 | 46XY | Endogenous:  *OCT3/4^+^, SOX2^+^, C-MYC^+^, REX1^+^, NANOG^+^*  Transgene:  *OCT3/4^-^, SOX2^-^, KLF4^-^, C-MYC^-^* | OCT3/4^+^  NANOG^+^  SSEA4^+^  TRA-1-60^+^ |
| UTA.00525.LQT2 | 46XY | Endogenous:  *OCT3/4^+^, SOX2^+^, C-MYC^+^, REX1^+^, NANOG^+^*  Transgene:  *OCT3/4^-^, SOX2^-^, KLF4^-^, C-MYC^-^* | OCT3/4^+^  NANOG^+^  SSEA4^+^  TRA-1-60^+^ |
